# CCA repair or ECA ligation—Which middle cerebral artery occlusion is better in the reperfusion mouse model?

**DOI:** 10.1002/ibra.12128

**Published:** 2023-08-18

**Authors:** Yue Hu, Zhen‐Hong Yang, Feng Yan, Shuang‐Feng Huang, Rong‐Liang Wang, Zi‐Ping Han, Jun‐Fen Fan, Yang‐Min Zheng, Ping Liu, Yu‐Min Luo, Si‐Jie Li

**Affiliations:** ^1^ Department of Neurology, Institute of Cerebrovascular Disease Research Xuanwu Hospital of Capital Medical University Beijing China; ^2^ Department of Emergency, Xuanwu Hospital Capital Medical University Beijing China; ^3^ Collaborative Innovation Center for Brain Disorders, Beijing Institute of Brain Disorders Capital Medical University Beijing China

**Keywords:** animal model, CCA repair, cerebral ischemia, stroke

## Abstract

A reliable animal model is essential for ischemic stroke research. The implications of the external carotid artery (ECA) transection or common carotid artery (CCA) ligation have been described. Thus, a modified animal model, the CCA‐repair model, has been established, and studies have shown that the CCA‐repair model has potential advantages over the CCA‐ligation model. However, whether the CCA‐repair model is superior to the ECA‐ligation model remains unclear. Sixty male C57BL/6 mice were randomly assigned to establish the CCA‐repair (*n* = 34) or ECA‐ligation (*n* = 26) models. Cerebral blood flow before middle cerebral artery occlusion (MCAO), immediately after MCAO and reperfusion were monitored and the operation duration, postoperative body weight, and food intake within 7 days, and the number of intraoperative and postoperative deaths within 7 days were recorded in the two models. Modified neurological severity scores and Bederson (0–5) scores were used to evaluate postoperative neurological function deficits on Days 1/3/5/7. 2,3,5‐Triphenyltetrazolium chloride staining was used to quantify lesion volume on Day 7 after the operation. We found the establishment of the CCA‐repair model required a longer total operation duration (*p* = 0.0175), especially the operation duration of reperfusion (*p* < 0.0001). However, there was no significant difference in body weight and food intake development, lesion volume and intragroup variability, neurological function deficits, mortality, and survival probability between the two groups. The CCA‐repair model has no significant advantage over the ECA‐ligation model. The ECA‐ligation model is still a better choice for focal cerebral ischemia.

## Graphical abstract

Two models were established: the common carotid artery‐repair model and the external carotid artery‐ligation model. The operation duration, postoperative body weight, and food intake within 7 days, neurological function deficits (modified neurological severity scores and Bederson [0–5] score) on Day 1/3/5/7 after operation, the number of intraoperative and postoperative deaths within 7 days and lesion volume on Day 7 were recorded in the two models. Which model is better for focal cerebral ischemia?

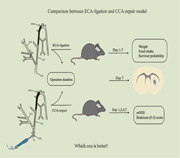

## 1 INTRODUCTION

With developing research on the pathogenesis, treatment, and prognosis of ischemic stroke, studies require a reliable and reproducible animal model that causes little harm to the experimental animals. The main cause of human ischemic stroke is the reduction or interruption of blood supply to the territory of the middle cerebral artery.^1^, ^2^ The intraluminal filament middle cerebral artery occlusion (MCAO) model is an important animal model of ischemic stroke. This model was first proposed by Longa et al.,^3^ and is simple, relatively noninvasive and reversible.^4^ Currently, the commonly used MCAO model includes two types: filament introduction via transection of the external carotid artery (ECA), that is, the ECA‐ligation model; and filament insertion via an incision in the common carotid artery (CCA), which is ligated during reperfusion, that is, the CCA‐ligation model.

With the extensive application of the MCAO model in mice, increasing problems such as unstable lesion volume with high intragroup variability, high mortality, and poor neurological function have appeared due to the disadvantages of the model itself, which has attracted the attention of researchers. Studies have shown that ECA transection interrupts the blood supply to the ECA territory, resulting in ischemia of the masticatory and swallowing muscles. This contributes to swallowing dysfunction, thus, decreasing the food intake of mice, leading to greater weight loss, impaired motor function, and poor neurological function.^5^ Another MCAO model with complete CCA ligation after reperfusion showed that blood flow reconstruction after reperfusion mainly depends on the circle of Willis.^6^ The high variation in the circle of Willis in mice^7^ may affect the restoration of blood flow in infarcted brain tissue after reperfusion.^8^ A modified MCAO model has been established based on the limitations of the two MCAO models; that is, the CCA is not ligated after reperfusion; rather, a muscle pad is attached using a fibrin sealant or microvascular suture to close the incision of the CCA.^6^, ^9^ The CCA‐repair model restores CCA blood supply after reperfusion, reducing reliance on the circle of Willis, which may be beneficial to the blood supply of the infarcted ipsilateral brain tissue. Trotman‐Lucas et al.^6^ compared a modified CCA‐repair model with a CCA‐ligation model in mice and found that repair of the CCA reduced lesion volume and variability within groups. This research shows that compared with the CCA‐ligation model, the CCA‐repair model reduces the confounding effects of the large lesion volume and high variability within groups of in vivo experiments, and it may be an alternative to the CCA‐ligation model.

An ideal MCAO mouse model requires a simple and reproducible operation, moderate neurological function deficit and lesion volume, minor intraoperative injury, and a high survival rate after MCAO. Although the CCA‐repair model has the potential advantages described above, it remains technically challenging^6^ and causes unstable stenosis and thrombosis in the CCA after surgery.^9^ STAIR group had recommended highly effective recanalization therapies, which are of utmost relevance to these models.^10^, ^11^ It is of great significance to select an appropriate ischemia‐reperfusion model for research. Whether the presumed advantages of CCA‐repair models are superior to those of the widely accepted ECA‐ligation model remains unknown. However, no study has been conducted to clarify this issue. In this study, the operation duration, body weight and food intake development, survival probability, lesion volume, and neurological function of the ECA‐ligation model were compared with those of the CCA‐repair model to provide evidence for the selection of MCAO models in the future.

## 2 MATERIALS AND METHODS

All mice were fed in a barrier system and were cared for by professional staff at the Chinese Center for Disease Control and Prevention. All animal experiments were approved by the Institutional Animal Care and Use Committee of Xuanwu Hospital of Capital Medical University (approval xw‐20220829‐2) on August 29, 2022. All experiments were carried out in accordance with the principles outlined in the National Institutes of Health (NIH) Guide for the Care and Use of Laboratory Animals.

### 2.1 Mouse model of MCAO

Sixty male C57BL/6 mice weighing 20–22 g (2‐month‐old, purchased from Si Pei Fu [Beijing] Biotechnology Co. Ltd.,) were numbered and were randomly assigned using a random number method. Because we are not familiar with the CCA‐repair model, 34 mice were assigned to the CCA‐repair model combined with the success rate in pre‐experimental, and 26 mice were assigned to the ECA‐ligation group. Anesthesia was induced by using an inhalation mixture of 5% isoflurane in nitrous oxide (N_2_O)/O_2_ (70%/30%). Anesthesia was maintained using a mixture of 2% isoflurane in N_2_O/O_2_. Rectal temperature was continuously monitored during the operation and a thermostatic heating pad was used to maintain the rectal temperature at 37°C ± 0.5°C. In the ECA‐ligation model, the ECA branches were cut after electrocoagulation and the right ECA was ligated with a 6.0 silk suture. A nylon monofilament (Doccol; filament size 6‐0, coating diameter 0.21 ± 0.01 mm, coating length 5–6 mm) was introduced into the intracranial internal carotid artery (ICA) through an incision made in the ligated ECA until its tip occluded the origin of the middle cerebral artery (MCA). In the CCA‐repair model, the right CCA was temporarily occluded using a microvascular clip without injury to the ECA or its branches. A nylon monofilament was introduced into the intracranial ICA through an incision made in the CCA until its tip occluded the origin of the MCA. After 45 min of MCAO, the mice were anesthetized, and the filament was withdrawn. In the ECA‐ligation group, the incision was closed directly using electrocoagulation after the filament was withdrawn. While in the CCA‐repair group, the incision on the CCA was carefully repaired by electric coagulation forceps to allow reperfusion to take place in the CCA.

### 2.2 Cerebral blood flow monitoring

The mouse was fixed to the stereotaxic instrument and anesthesia was maintained using a mixture of 2% isoflurane in N_2_O/O_2_. An incision was made along the midline of the scalp to explore the skull. The skull is kept moist with 0.9% sterile saline. A laser speckle probe (LDF, PeriFlux System 5000; Perimed) was positioned on the center of the skull. The cerebral blood flow was recorded before MCAO and immediately after cerebral ischemia and reperfusion; each recording lasted for about 20 s. The cerebral blood flow was analyzed according to the cerebral blood flow imaging and the decreasing rate of the cerebral blood flow on the infarction side immediately after cerebral ischemia and reperfusion was also obtained.

### 2.3 Operation duration record

The time required to induce ischemia was calculated as the time from the start of maintenance anesthesia until the occlusion of the MCA's origin. The operation duration of reperfusion was recorded from anesthesia after 45‐min MCAO to reperfusion. The length of both the ischemia‐inducing and reperfusion operations was the total operation time.

### 2.4 Body weight and food intake record

The identical feeding protocol was used for all mice, and one mouse per cage. Within 3 days of MCAO and 7 days following MCAO, each mouse was weighed at the same time each day. On Day 4 before to MCAO, each mouse received the baseline feeding amount, which was set at 30 g. From Day 3 before MCAO to Day 7 after MCAO, the 24‐h food excess was weighed at the same time every day. The 24‐h food surplus was weighed at the same time every day from Day 3 before MCAO to Day 7 after MCAO. The 24‐h food intake of each mouse was calculated as the baseline value of the feed minus the food surplus after 24 h. After weighing the 24‐h food surplus, it was changed to the baseline feeding value (30 g) to reduce experimental errors.

### 2.5 Neurological assessment and lesion volume measurement

All mice were neurologically examined on Day 1/3/5/7 after MCAO according to the modified neurological severity scores (mNSS)^12^, ^13^ and Bederson (0–5) scores^14^, ^15^ (0: no deficit; 1: forelimb flexion; 2: decreased resistance to lateral push; 3: unidirectional circling; 4: longitudinal spinning; 5: no movement). 7d△mNSS (mNSS_Day1_ − mNSS_Day7_) and 7d△Bederson (0–5) score (Bederson_Day1_ − Bederson_Day7_) were then recorded. The animals were killed on Day 7 after MCAO using an intraperitoneal injection of 0.1 mL 10% chloral hydrate. The removed brains were sectioned into 8 × 1‐mm coronal slices and stained using 2% 2,3,5‐triphenyltetrazolium chloride (TTC) (SAITONG; Beijing Jinming Biotechnology Co. Ltd.) to quantify the lesion volume. Lesion volume was calculated using ImageJ software (NIH).

### 2.6 Statistical analysis

SPSS (version 22.0; IBM Corp.), GraphPad Prism 8.2.1 (GraphPad Software), and R software (version 4.1.1) were used for the statistical analyses. Statistical significance was set at *p* < 0.05. Kolmogorov–Smirnov test was used to assess the normal distribution of data. Continuous variables conforming to a normal distribution are reported as the mean ± SD. Variables not conforming to a normal distribution assumption are represented as the median (interquartile range, *Q*_1_–*Q*_3_). Comparison between the two groups was tested using the unpaired *t*‐test or Mann–Whitney. Comparison between multiple groups was performed using analysis of variance on ranks. The intragroup variability in lesion volume was compared using the *F*‐test. The Kaplan–Meier curve was used to analyze survival probability.

## 3 RESULTS

### 3.1 Stability evaluation of the CCA‐repair and the ECA ligation model

**Figure 1:**
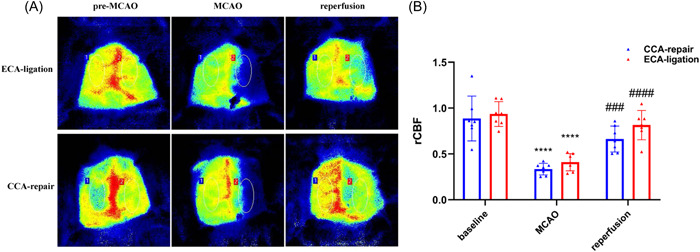
Comparison in cerebral blood flow change between CCA‐repair group and ECA‐ligation group immediately after cerebral ischemia and reperfusion. (A) Cerebral blood flow imaging in the CCA‐repair group and ECA‐ligation group (red means abundant blood flow; yellow means moderate blood flow; green and blue mean low blood flow. (B) Cerebral blood flow before MCAO, immediately after cerebral ischemia and reperfusion between the CCA‐repair group and the ECA‐ligation group. CCA‐repair, *n* = 7; ECA‐ligation, *n* = 7; ****p < 0.0001: comparison with baseline; ^###^ *p* < 0.001, ^####^ *p* < 0.0001: comparison with MCAO. CCA, common carotid artery; ECA, external carotid artery; MCAO, middle cerebral artery occlusion; rCBF, regional cerebral blood flow. [Color figure can be viewed at wileyonlinelibrary.com]

We recorded the operative field and it can be roughly seen that the blood flow is restored (Figure 1A). To ensure the success of the model, laser speckle imaging was further used to monitor the cerebral blood flow during ischemia and reperfusion in the two models. We analyzed the cerebral blood flow immediately after cerebral ischemia and reperfusion, we found cerebral blood flow decreased significantly after MCAO immediately compared with baseline (CCA‐repair model, *n* = 7, *p* < 0.0001; ECA‐ligation model, *n* = 7, *p* < 0.0001; Figure 1B) and cerebral blood flow was significantly restored after reperfusion compared with post‐MCAO (CCA‐repair model, *n* = 7, *p* < 0.001; ECA‐ligation model, *n* = 7, *p* < 0.0001; Figure 1B) in both groups. No difference was found in cerebral blood flow at baseline (*p* = 0.6533), immediately after MCAO (*p* = 0.1049), and reperfusion (*p* = 0.0837) between the two models (Figure 1B). These results indicate that the two models we established are stable.

### 3.2 The CCA‐repair model required a longer operation duration than the ECA‐ligation model

**Figure 2:**
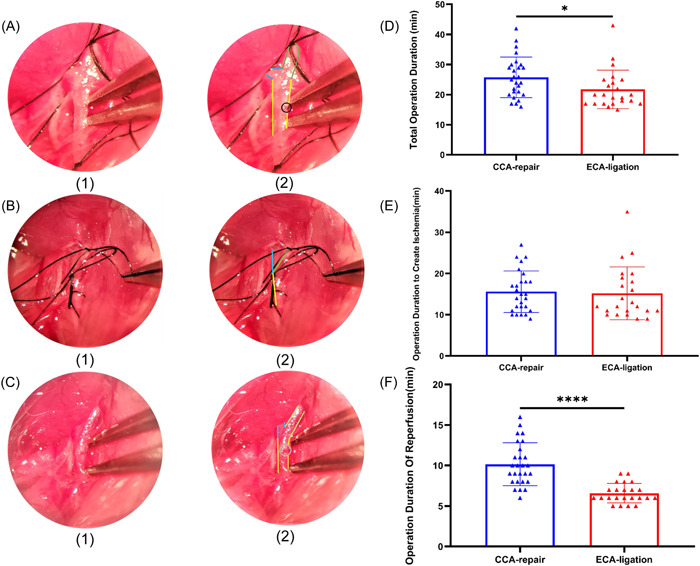
Photomicrographs in CCA‐repair model and comparison of operation duration in CCA‐repair group and ECA‐ligation group. (A) Photomicrographs show incision, (B) filament insertion and incision repair (C) (1) represents original images; (2) represents original images with marked structure. Blue means internal carotid artery; green means external carotid artery; yellow means common carotid artery. (D) Overall operation duration was significantly longer in the CCA‐repair group than in the ECA‐ligation group *p = 0.0175. (E) No significant difference in operation duration to create ischemia in the two groups. (F) Operation duration of reperfusion was significantly longer in the CCA‐repair group than in the ECA‐ligation group ****p < 0.0001. CCA, common carotid artery; ECA, external carotid artery. [Color figure can be viewed at wileyonlinelibrary.com]

We recorded the surgical procedure of the CCA‐repair MCAO model, including the operative field (Figure 2A–C) and duration (Figure 2D–F). The incision of the CCA‐repair model needs to be repaired during reperfusion, which takes longer than direct coagulation of the ECA transection (CCA‐repair group: 10.1 ± 2.6 min; ECA‐ligation group: 6.6 ± 1.2 min) (*p* < 0.0001; Figure 2F). Regarding the total operation duration in the two groups, we found that the total operation duration in the CCA‐repair group (25.7 ± 6.7 min) was significantly longer than that in the ECA‐ligation group (21.7 ± 6.4 min) (*p* = 0.0175; Figure 2D). However, the operation duration to create ischemia (CCA‐repair group: 15.6 ± 5.0 min; ECA‐ligation group: 15.2 ± 6.4 min) showed no significant difference between the two groups (*p* = 0.5067; Figure 2E). These results suggest that the duration of CCA repair surgery is longer, which is equivalent to the longer duration of anesthesia in mice. It may also affect lesion volume and cause neurological function deficit.^16^

### 3.3 There was no significant difference between the CCA‐repair group and the ECA‐ligation group in body weight development

**Figure 3:**
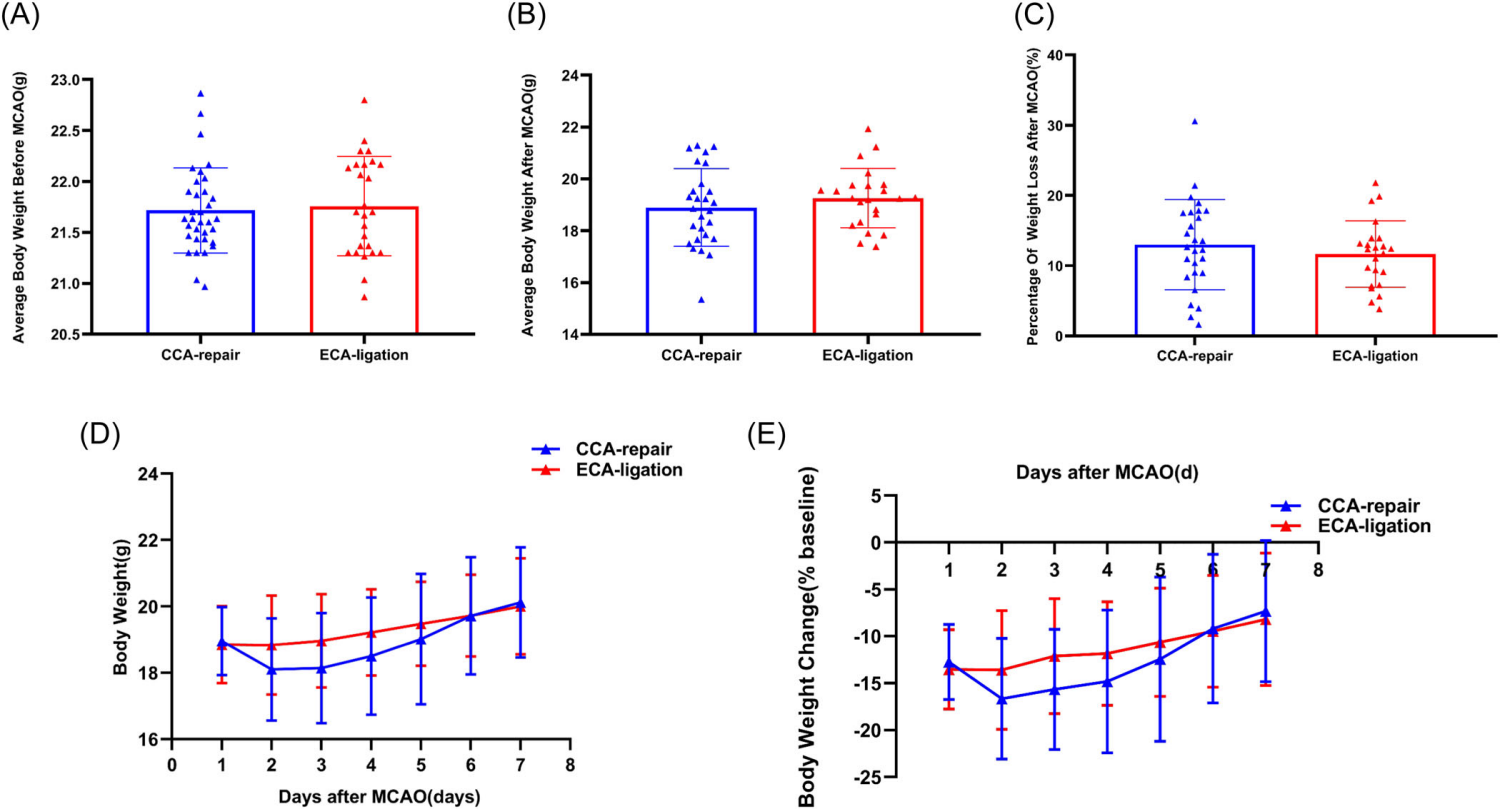
Comparison of body weight development between CCA‐repair group and ECA‐ligation group. (A) Average body weight within 3 days before MCAO. (B) Average body weight within 7 days after MCAO. (C) Percentage of weight loss on Day 7 post‐MCAO. (D) Body weight and (E) body weight change within 7 days after MCAO. CCA, common carotid artery; ECA, external carotid artery; MCAO, middle cerebral artery occlusion. [Color figure can be viewed at wileyonlinelibrary.com]

There was no significant difference in the average body weight within 3 days preoperatively between the CCA‐repair group (21.7 ± 0.4 g) and the ECA‐ligation group (21.8 ± 0.5 g) (*p* = 0.7211; Figure 3A), which ensured comparability of body weight between the two groups. The average body weight was slightly lower in the CCA‐repair group (18.9 ± 1.5 g) than in the ECA‐ligation group (19.3 ± 1.1 g) within 7 days after MCAO; however, the difference was not significant (*p* = 0.3500; Figure 3B). Further, the percentage of weight loss within 7 days after MCAO in the CCA‐repair group (12.9981 ± 6.43583%) was slightly higher than that in the ECA‐ligation group (11.6510 ± 4.72884%); however, the difference was not significant (*p* = 0.4104; Figure 3C). The lowest weight was observed on Day 2 after MCAO in both the ECA‐ligation and CCA‐repair groups (Figure 3D) and weight loss was greater in the CCA‐repair group (Figure 3D,E). The body weight of the ECA‐ligation group was higher than those of the CCA‐repair group on Days 2 (*p* = 0.0949), 3 (*p* = 0.0698), 4 (*p* = 0.1157), and 5 (*p* = 0.3458); however, there was no significant difference (Figure 3D). Further, the body weight of the ECA‐ligation group was lower than those of the CCA‐repair group on Days 1 (*p* = 0.7405) and 7 (*p* = 0.5079) after MCAO; however, there was still no significant difference (Figure 3D). The body weight of the CCA‐repair group increased faster on Days 3 (*p* = 0.0521), 4 (*p* = 0.1242), 5 (*p* = 0.4051), and 6 (*p* = 0.6122) after MCAO; however, no significant difference was found between the two groups (Figure 3E). All the above results indicated that body weight development between the CCA‐repair group and the ECA‐ligation group were not significantly different within 7 days after MCAO, suggesting that the CCA‐repair model did not ameliorate the ECA‐ligation model for greater weight loss after MCAO.

### 3.4 There was no significant difference in food intake development between the CCA‐repair group and ECA‐ligation group

**Figure 4:**
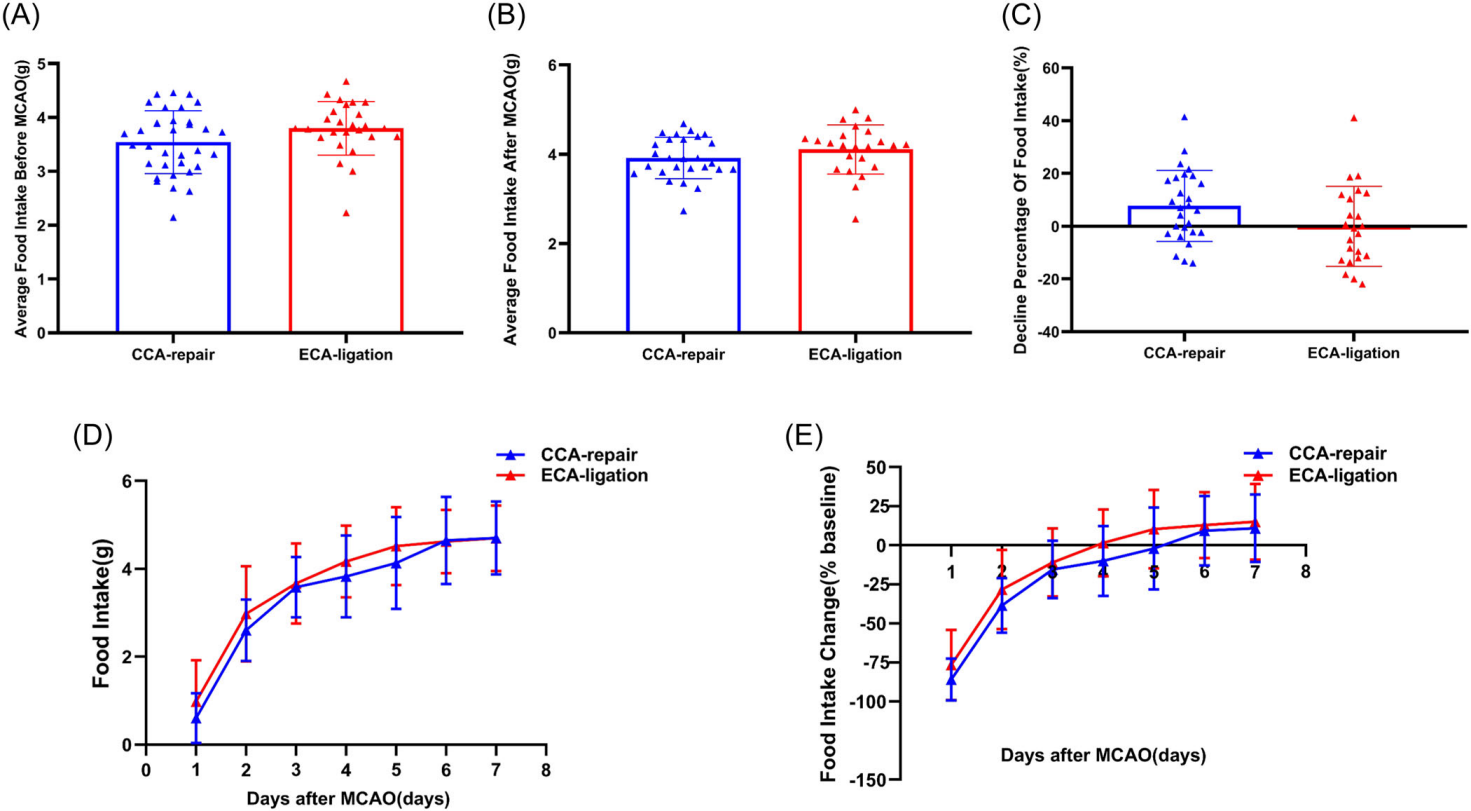
Comparison of food intake development between the CCA‐repair group and ECA‐ligation group. (A) Average food intake within 3 days before MCAO. (B) Average food intake within 7 days after MCAO. (C) Decline percentage of food intake on Day 7 post‐MCAO. (D) Food intake and (E) food intake change within 7 days after MCAO. CCA, common carotid artery; ECA, external carotid artery; MCAO, middle cerebral artery occlusion. [Color figure can be viewed at wileyonlinelibrary.com]

There was no significant difference between the average food intake in the CCA‐repair group (3.5 ± 0.6 g) and ECA‐ligation group (3.8 ± 0.5 g) (*p* = 0.0767; Figure 4A) within 3 days before MCAO, which ensured comparability of food intake between the two groups. The average food intake in the ECA‐ligation group (4.1 ± 0.5 g) was slightly higher than that in the CCA‐repair group (3.9 ± 0.5 g); however, there was no significant difference between the two groups (*p* = 0.1869; Figure 4B). The percentage decline in food intake in the CCA‐repair group (7.674 ± 13.2281%) was slightly higher than that in the ECA‐ligation group (−0.0573 ± 14.8388%); however, no significant difference was found between the two groups (*p* = 0.0624; Figure 4C). The food intake in the ECA‐ligation group was higher than that in the CCA‐repair group on Days 1–5 after MCAO (Day 1, *p* = 0.3571; Day 2, *p* = 0.1475; Day 3, *p* = 0.2345; Day 4, *p* = 0.1808; Day 5, *p* = 0.1753), with no significant difference between the two groups (Figure 4D). However, the food intake in the two groups was approximately equal on Days 6 and 7 after MCAO (Figure 4D). Both groups showed the greatest decrease in food intake on Day 1 and presented a gradually increasing trend until Day 7 (Figure 4E). Compared to the baseline value, the change in food intake from Day 1 to 7 after MCAO in the ECA‐ligation group was lower than that in the CCA‐repair group (Day 1, *p* = 0.3165; Day 2, *p* = 0.0997; Day 3, *p* = 0.1580; Day 4, *p* = 0.0712; Day 5, *p* = 0.1523; Day 6, *p* = 0.5655; and Day 7, *p* = 0.5180), but no statistical difference was found between the two groups (Figure 4E). The ECA‐ligation group returned to the baseline earlier than the CCA‐repair group (Figure 4E). Both groups exceeded the baseline value on Day 7 after MCAO (Figure 4E). The above results showed no significant difference in food intake development between the CCA‐repair and ECA‐ligation groups within 7 days after MCAO, indicating that the CCA‐repair model did not improve the inadequacy of food intake reduction in the ECA‐ligation model.

### 3.5 There was no significant difference in mortality and survival probability between the CCA‐repair group and ECA‐ligation group

The 60 mice were randomly divided into two groups: 34 mice in the CCA‐repair group and 26 mice in the ECA‐ligation group. In the CCA‐repair group, two mice died after filament withdrawal. Two mice died on Days 3 and 4 and one on Day 5 after MCAO. The intraoperative mortality was 5.88%, and postoperative mortality was 6.25%, 6.67%, and 3.57% on Days 3, 4, and 5, respectively. The overall postoperative mortality was 15.62%, and the overall mortality during and after MCAO was 20.59%. Finally, 27 mice were left for statistical analysis in the CCA‐repair group.

In the ECA‐ligation group, no deaths occurred during surgery. One mouse each died on Days 3, 4, and 5 after MCAO. The intraoperative mortality rate was 0%, and the postoperative mortality rates were 3.84%, 4.00%, and 4.16% on Days 3, 4, and 5, respectively. The overall postoperative mortality was 11.54%, and the overall mortality during and after MCAO was 11.54%. Finally, 23 mice were left for statistical analysis in the ECA‐ligation group.

**Figure 5:**
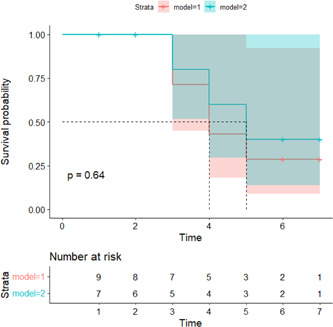
Comparison of survival probability within 7 days post‐MCAO between CCA‐repair group and ECA‐ligation group. Model 1 = CCA‐repair model, *n* = 5; model 2 = ECA‐ligation model, *n* = 3. CCA, common carotid artery; ECA, external carotid artery; MCAO, middle cerebral artery occlusion. [Color figure can be viewed at wileyonlinelibrary.com]

No significant differences were found in intraoperative mortality (*p* = 0.208) and mortality on Days 3 (*p* = 0.681), 4 (*p* = 0.665), and 5 (*p* = 0.911) after MCAO between the two groups. In addition, there was no significant difference in overall postoperative mortality (*p* = 0.654) and in total preoperative and postoperative mortality between the two groups (*p* = 0.351). The K–M analysis between the two groups showed no significant difference in the downward trend of the curves (Figure 5; *p* = 0.6396); that is, there was no statistical difference in the survival probability between the two groups after MCAO.

These results suggest that the CCA‐repair model did not ameliorate the high mortality caused by ECA transection in the ECA‐ligation model.

### 3.6 There was no significant difference in lesion volume and intragroup variability between the CCA‐repair group and ECA‐ligation group

**Figure 6:**
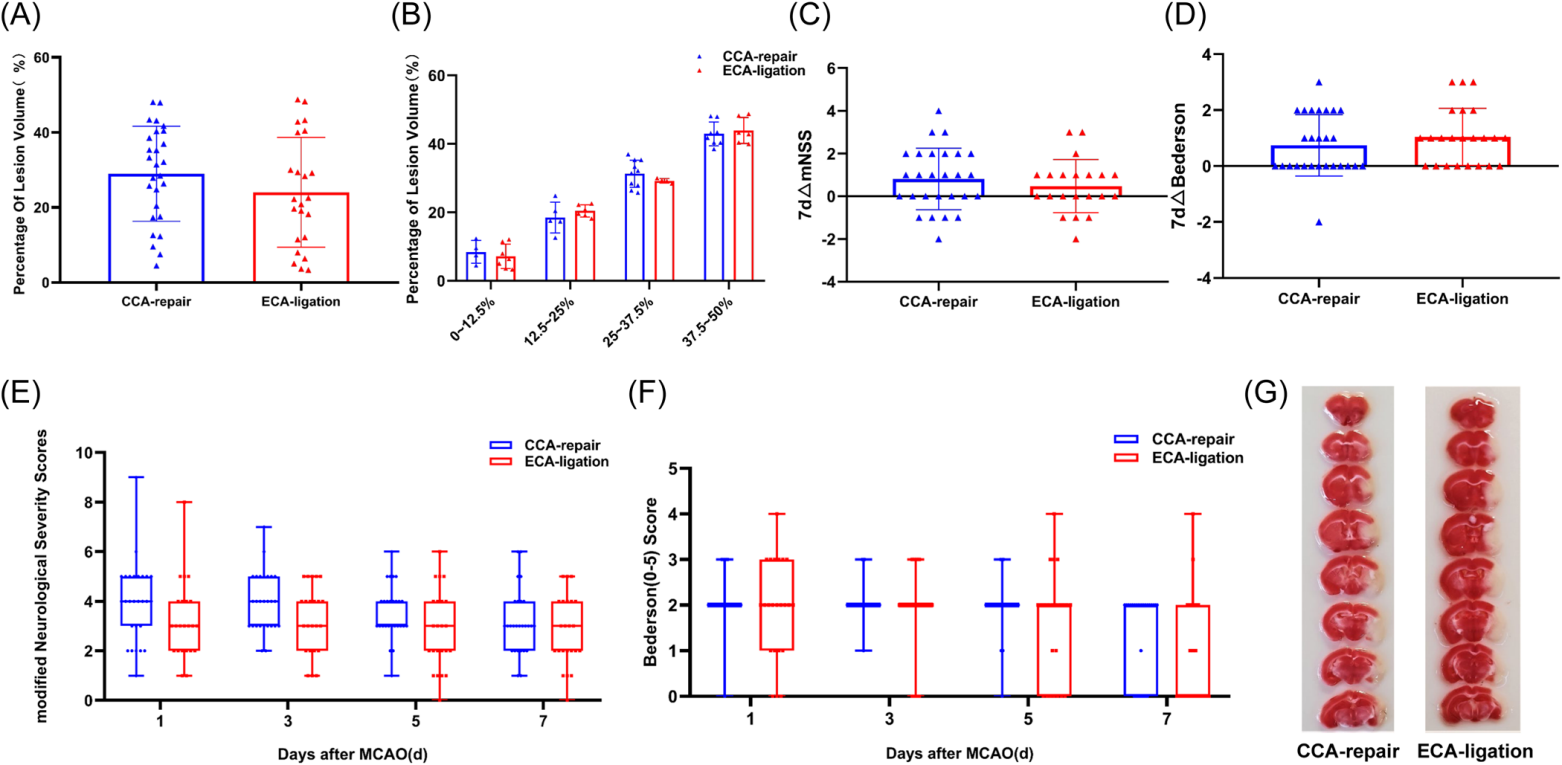
Comparison of lesion volume and neurological function deficit between CCA‐repair group and ECA‐ligation group. (A) Percentage of lesion volume on Day 7 post‐MCAO. (B) Percentage of lesion volume with distinction of grade in 0%–12.5%, 12.5%–25%, 25%–37.5%, 37.5%–50% on Day 7 post‐MCAO. (C) 7d△mNSS (mNSS_Day1_ − mNSS_Day7_) and (D) 7d△Bederson (0–5) score (Bederson_Day1_ − Bederson_Day7_). Box and whisker plots demonstrating mNSS (E) and Bederson (0–5) score (F) on Day 1/3/5/7 post‐MCAO. The horizontal line in each box represents the median with the box representing the interquartile range (*Q*_3_–*Q*_1_) and the whiskers representing the total range of the data (maximum to minimum). (G) TTC staining of brain tissue on Day 7 post‐MCAO. CCA, common carotid artery; ECA, external carotid artery; MCAO, middle cerebral artery occlusion; mNSS, modified neurological severity scores; TTC, 2,3,5‐triphenyltetrazolium chloride. [Color figure can be viewed at wileyonlinelibrary.com]

We compared lesion volume on Day 7 after MCAO between the CCA‐repair and ECA‐ligation groups. TTC staining of the brain sections showed that the infarct sites in the two groups were the same, both in the right cerebral cortex and basal ganglia (Figure 6G). First, we compared lesion volumes between the two groups. The lesion volume in the CCA‐repair group (28.9981 ± 12.69814%) was larger than that in the ECA‐ligation group (24.0348 ± 14.65661%), with no statistically significant difference between the two groups (*p* = 0.2056; Figure 6A). Moreover, there was no significant difference in the variation of lesion volume between the two groups (CCA‐repair group: *n* = 27; ECA‐ligation group: *n* = 23. *p* = 0.4802, *F*‐test).

We then compared the four different levels of lesion volume: 0%–12.5% (*p* = 0.558025), 12.5%–25% (*p* = 0.364286), 25%–37.5% (*p* = 0.335963) and 37.5%–50% (*p* = 0.627972); there was no statistically significant difference in the lesion volume between the two groups at any level. The lesion volume was 0%–12.5% (CCA‐repair group: *n* = 4; ECA‐ligation group: *n* = 7; *p* = 0.9901, *F*‐test), 12.5%–25% (CCA‐repair group, *n* = 5; ECA‐ligation group, *n* = 6. *p* = 0.0638, *F*‐test), and 37.5%–50% (CCA‐repair group: *n* = 8; ECA‐ligation group: *n* = 6. *p* = 0.8159, *F*‐test), with no statistically significant difference in variability within the groups; however, the lesion volume was 25%–37.5% (CCA repair group: *n* = 10; ECA ligation group: *n* = 4. *p* = 0.0134, *F*‐test), with significant differences in variability within the groups, and greater intragroup difference in the CCA‐repair group (Figure 6B).

These results indicated that the CCA‐repair model did not reduce the lesion volume and variability within the group compared to the ECA‐ligation model, which also suggested that the presumed advantages of the CCA‐repair model over the CCA‐ligation model were not superior to those of the ECA‐ligation model.

### 3.7 There was no significant difference in neurological function deficit between the CCA‐repair group and ECA‐ligation group

The mNSS indicated that the neurological function deficits in the two groups were generally mild injury (0–6 points) within 7 days after MCAO, and the neurological function deficit in the ECA‐ligation group gradually increased from Day 1 to 3, whereas the neurological function deficit in the CCA‐repair group remained almost unchanged from Day 1 to 3 (Figure 6E). Neurological function gradually recovered from Day 3 after MCAO in both groups (Figure 6E). Although neurological function deficit was greater in the CCA‐repair group, there was no significant difference between the two groups (Day 1, *p* = 0.0590; Day 3, *p* = 0.1131; Day 5, *p* = 0.0675; Day 7, *p* = 0.5764; Figure 6E). We analyzed the recovery of mNSS between the two groups and we found most mice showed neurological function recovery within 7 days after MCAO, and no significant difference was found between the two groups (*p* = 0.4193; Figure 6C).

Bederson (0–5) scores showed that the neurological function deficit was the most severe in both groups on Day 1, mostly with mild to moderate injury (1–3 points) (Figure 6F). The neurological function of both groups gradually recovered from Day 1 to 7, and mild injury (1–2 points) was observed in both groups on Day 7 after MCAO (Figure 6F). Although there was a more severe neurological deficit in the CCA‐repair group than in the ECA‐ligation group, there was no statistically significant difference between the two groups (Day 1, *p* = 0.5445; Day 3, *p* = 0.6691; Day 5, *p* = 0.1561; Day 7, *p* = 0.0783, Figure 6F). We also analyzed recovery of Bederson (0–5) scores between the two groups and almost all mice showed neurological function recovery within 7 days after MCAO, and no significant difference was found between the two groups (*p* = 0.3513; Figure 6D).

Although there were some differences in the evaluation of postoperative neurological function deficits between the two groups using two different scoring systems, both scoring systems indicated that the injury in the two groups was more serious on Days 1 and 3, and gradually recovered until Day 7 after MCAO. These results indicated that the CCA‐repair model did not reduce neurological injury in mice after MCAO.

## 4 DISCUSSION

Animal models are important tools to understand the pathophysiology of ischemic stroke. Therefore, it is crucial to select a reliable animal model for ischemic stroke. The modified CCA‐repair model has the advantage of reducing lesion volume and variability within groups compared to the CCA‐ligation model. In the present study, we confirmed the CCA‐repair model we established was a stable cerebral ischemia/reperfusion model and we described for the first time that the CCA‐repair model has no significant difference in body weight and food intake development, lesion volume and intragroup variability, neurological function, mortality, and survival probability compared with the ECA‐ligation model, but the CCA‐repair model required a longer operation duration. These results clarify, for the first time, that the CCA‐repair model has no significant advantage over the ECA‐ligation model.

The translational research of ischemic stroke requires stable cerebral ischemic animal models. In our study, the laser speckle was used to demonstrate the stability of the two models, and mainly to demonstrate the reperfusion of blood flow after the incision was repaired in the CCA‐repair model. The results showed reperfusion was successful and stable in the two models, although cerebral blood flow was slightly lower in the CCA‐repair model. Studies have shown thrombosis was detected in the CCA‐repair model, which can partly explain reduced blood supply in the CCA‐repair model after reperfusion.^9^ In our study, the intersubject variability may have an effect on the difference in the reperfusion between the two models. Therefore, larger sample sizes may be needed to confirm this.

An ideal animal model requires avoiding over‐anesthesia of the experimental animal to reduce confounding factors affecting lesion formation. In our study, compared with the ECA‐ligation model, the CCA‐repair model required a longer operation duration to ensure blood flow recanalization of the CCA, mainly because it took longer to repair the incision. To ensure blood flow recanalization in the CCA, the incision should be carefully repaired after filament withdrawal, which is technically challenging for surgeons. Previous research has reported a CCA‐repair model that depicted a muscle pad attached using a fibrin sealant or a microvascular suture to close the incision of the CCA during reperfusion.^9^ In the CCA‐repair model established in our study, the incision was repaired using electrocoagulation. The method we used to repair the incision is simpler than that of the others but may require more technical expertize by the surgeon. In addition, the longer operative duration was equivalent to that in mice undergoing longer periods of anesthesia. Delayed or lasting effects of anesthetics have been shown to have significant effects on both clinical and experimental neuronal injury.^17^ Therefore, the longer duration of anesthesia required in the CCA‐repair model may be a critical confounding factor affecting neurological recovery and lesion volume in mice.

Ligation damages the ECA and its branch arteries, resulting in masticatory and swallowing ischemia and dysfunction, reduced food intake, and increased body weight loss. However, the CCA‐repair model did not damage the ECA territory. Does the CCA‐repair model improve the inadequacy of the ECA‐ligation model? Our study showed no significant differences in body weight or food intake development between the two groups within 7 days after MCAO, indicating that the CCA‐repair model could not ameliorate food intake reduction and greater weight loss in the ECA‐ligation model. Trotman‐Lucas et al.^6^ compared body weight loss between the ECA‐ligation combined with analgesia and the CCA‐repair group (ECA‐unligation) combined with no analgesia with the same time period as MCAO. Their results showed a trend towards improved body weight loss in the CCA‐repair and ECA‐ligation models within 2 days after MCAO, but there was no significant difference between the two groups. Although their results were inconsistent with ours, their research could not determine whether the weight loss was related to CCA repair or analgesia. In addition, they compared weight changes within 2 days after MCAO, which did not reflect the overall development of weight loss in mice. Although the CCA‐repair model did not damage the artery that supplied the masticatory muscles, Dittmar et al.^9^ observed the masticatory muscles of rats after CCA repair and found masticatory lesions using MRI. They found thrombosis adhering to the walls of the CCA in histological sections. Therefore, they speculated that masticatory lesions might be caused by detachment of the thrombus, which blocks the blood flow supplying the masticatory muscle, resulting in masticatory muscle ischemia.^9^ In our study, although the incision was selected far away from the bifurcation of the ICA and ECA, and fibrin sealant, a coagulant, was not used; thrombosis may also occur at the electrocoagulation site due to hemodynamic changes in the CCA‐repair model. Cullins et al.^18^ confirmed that the swallowing function is impaired and tongue force is reduced in rats after MCAO. Wessig et al.^19^ found that MRI and electrophysiological signals changed after muscle denervation. Therefore, in addition to the intraoperative injury of the ECA territory, which may influence postoperative food intake and body weight, neurological injury caused by the stroke itself also leads to masticatory and swallowing dysfunctions in experimental animals. Furthermore, when Dittmar et al.^5^ proposed that ischemic injury of the masticatory and swallowing muscles is caused by ECA ligation, they also pointed out that animals with marked collateral blood supply to the ECA territory are probably less susceptible to ischemia after ECA ligation. In conclusion, both the CCA‐repair and ECA‐ligation models may have masticatory lesions as well as masticatory and swallowing dysfunction caused by neurological injury after MCAO. Therefore, there was no significant difference in body weight or food intake between the two groups after MCAO. To calculate the food intake of each mouse, a feeding model with one mouse per cage was used. Mice are typically social animals; therefore, they consume more food and grow faster when kept in groups than when kept alone.^20^, ^21^ Therefore, compared with group feeding, our feeding model may affect the food intake and body weight of mice, but has little impact on our experimental results.

A successful animal model requires a moderate and stable lesion volume. The different parameters of the model itself, including occlusion and recovery time, the filament and anesthetic used, and differences in the surgical technique as well as parameters of the animals, is one of the most important outcome parameters which impact lesion volume.^7^, ^22^ In our study, 45‐min occlusion time is beneficial to the animal recovery after the operation and streamlines the experimental process. The CCA was ligated after filament withdrawal in the CCA‐ligation model, so reperfusion of the infarcted ipsilateral brain tissue is mainly reliant on the circle of Willis.^6^ However, owing to the high variability in the circle of Willis between mice, variability in PCA contributes to variations in lesion volume.^23^, ^24^ As previously mentioned, Trotman‐Lucas et al.^6^ demonstrated that the CCA‐repair model reduced lesion volume and intragroup variability compared with the CCA‐ligation model. Is there any difference in lesion volume and intragroup variability between the CCA‐repair and ECA‐ligation models? Our study showed that there was no significant difference in lesion volume and intragroup variability between the two groups on Day 7 after MCAO, and the intragroup variability in lesion volume in 0%–12.5%, 12.5%–25%, and 37.5%‐50% have no significant difference. However, the intragroup variability of lesion volume in the 25%–37.5% range was significantly different between the two groups (*p* = 0.0134), which might have been due to the significant difference in the number of mice in this lesion volume interval between the two groups. The CCA was recanalized after filament withdrawal in both the ECA‐ligation and CCA‐repair models, which reduced the reliance on the circle of Willis for reperfusion.

In addition to moderate lesion volume with low variability, stable neurological impairment, and low mortality are required for successful animal models. Thus, we compared the mortality and survival probability of the CCA‐repair and ECA‐ligation groups within 7 days after MCAO. Moreover, we chose mNSS and Bederson scores on Days 1/3/5/7 after MCAO to evaluate neurological function deficits, which had been confirmed to have better test validities^25^ and were simple enough to carry out. However, no significant differences were found between the two groups. Studies have shown that poststroke mortality in mice is affected by insufficient food intake and drinking.^26^ As previously mentioned, the feeding model of one mouse per cage may influence food intake. Therefore, the adopted feeding model may have resulted in a higher mortality rate in mice after MCAO. In our study, the neurological function deficit in both the CCA‐repair and ECA‐ligation groups was mild‐to‐moderate within 7 days after MCAO and gradually recovered to mild injury on Day 7, which is the ideal degree of neurological injury.

## 5 CONCLUSION

We confirmed both the CCA‐repair model and the ECA‐ligation model are stable cerebral ischemia/reperfusion models. However, compared with the ECA‐ligation model, the CCA‐repair model does not simplify the operation procedure, which requires greater technical expertize and a longer operation duration. The CCA‐repair model did not ameliorate the reduced food intake and increased weight loss caused by ECA ligation. Furthermore, the CCA‐repair model did not reduce the lesion volume and variability within groups compared to the ECA‐ligation model. In addition, the degree of neurological injury in both the CCA‐repair and ECA‐ligation models is required in the ideal model. In conclusion, the CCA‐repair model does not have any potential advantages over the ECA‐ligation model, and the ECA‐ligation model remains a better transformation model for focal cerebral ischemia.

## AUTHOR CONTRIBUTIONS

Yue Hu and Zhen‐Hong Yang conducted the study design, experiments, data analysis, and manuscript preparation. Yu‐Min Luo and Zhen‐Hong Yang designed and managed the study. Other authors reviewed and edited the manuscript.

## CONFLICT OF INTEREST STATEMENT

The authors declare no conflict of interest.

## ETHICS STATEMENT

All animal experiments were approved by the Institutional Animal Care and Use Committee of Xuanwu Hospital of Capital Medical University (approval xw‐20220829‐2) on August 29, 2022. All experiments were carried out in accordance with the principles outlined in the National Institutes of Health (NIH) Guide for the Care and Use of Laboratory Animals.

## Data Availability

Data supporting the findings of this study are available from the corresponding authors.
